# *SELENOI* Functions as a Key Modulator of Ferroptosis Pathway in Colitis and Colorectal Cancer

**DOI:** 10.1002/advs.202404073

**Published:** 2024-05-17

**Authors:** Xin Huang, Xu Yang, Mingxin Zhang, Tong Li, Kongdi Zhu, Yulan Dong, Xingen Lei, Zhengquan Yu, Cong Lv, Jiaqiang Huang

**Affiliations:** ^1^ Key Laboratory of Precision Nutrition and Food Quality Department of Nutrition and Health China Agricultural University Beijing 100193 China; ^2^ Beijing Advanced Innovation Center for Food Nutrition and Human Health Department of Nutrition and Health China Agricultural University Beijing 100193 China; ^3^ College of Biological Sciences China Agricultural University Beijing 100193 China; ^4^ College of Veterinary Medicine China Agricultural University Beijing 100193 China; ^5^ Department of Animal Science Cornell University Ithaca NY 14853 USA

**Keywords:** colorectal cancer, ether lipids, ferroptosis, intestinal regeneration, *selenoi*

## Abstract

Ferroptosis plays important roles both in normal physiology and multiple human diseases. It is well known that selenoprotein named glutathione peroxidase 4 (GPX4) is a crucial regulator for ferroptosis. However, it remains unknown whether other selenoproteins responsible for the regulation of ferroptosis, particularly in gut diseases. In this study, it is observed that Selenoprotein I (*Selenoi*) prevents ferroptosis by maintaining ether lipids homeostasis. Specific deletion of *Selenoi* in intestinal epithelial cells induced the occurrence of ferroptosis, leading to impaired intestinal regeneration and compromised colonic tumor growth. Mechanistically, *Selenoi* deficiency causes a remarkable decrease in ether‐linked phosphatidylethanolamine (ePE) and a marked increase in ether‐linked phosphatidylcholine (ePC). The imbalance of ePE and ePC results in the upregulation of phospholipase A2, group IIA (Pla2g2a) and group V (Pla2g5), as well as arachidonate‐15‐lipoxygenase (Alox15), which give rise to excessive lipid peroxidation. Knockdown of PLA2G2A, PLA2G5, or ALOX15 can reverse the ferroptosis phenotypes, suggesting that they are downstream effectors of *SELENOI*. Strikingly, GPX4 overexpression cannot rescue the ferroptosis phenotypes of *SELENOI*‐knockdown cells, while *SELENOI* overexpression can partially rescue GPX4‐knockdown‐induced ferroptosis. It suggests that *SELENOI* prevents ferroptosis independent of GPX4. Taken together, these findings strongly support the notion that *SELENOI* functions as a novel suppressor of ferroptosis during colitis and colon tumorigenesis.

## Graphical abstract

Specific deletion of *Selenoi* in intestinal epithelial cells induces the occurrence of ferroptosis, leading to impaired intestinal regeneration and compromised colonic tumor growth. Mechanistically, *Selenoi* deficiency causes a remarkable decrease in ether‐linked phosphatidylethanolamine (ePE) and a marked increase in ether‐linked phosphatidylcholine (ePC). The imbalance of ePE and ePC results in excessive lipid peroxidation.

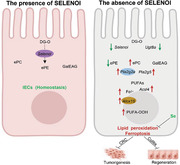

## 1 Introduction

A major cause of cancer‐related deaths globally is colorectal cancer (CRC),^[^ ^1^ ^]^ which is usually caused by a combination of genetic and environmental risk factors that lead to hyperplastic lesions and ultimately carcinoma.^[^ ^2^ ^]^ Colitis‐associated colon cancer (CAC) is one of the most aggressive subtypes of CRC, and as a result of prolonged chronic inflammation and develops in patients with inflammatory bowel disease (IBD).^[^ ^2^ ^]^ Destruction of epithelial cells and accumulation of inflammatory cells within the submucosa are common features of IBD.^[^ ^3^, ^4^ ^]^ This damage initiates a complicated repair program that activates epithelial cell proliferation and promotes the regeneration of impaired epithelium. When intestinal epithelial damage cannot be repaired, chronic inflammation‐induced cell proliferation may promote tumorigenesis,^[^ ^3^ ^]^ which is tough to cure with a high mortality rate.^[^ ^5^ ^]^

Ferroptosis is a novel iron‐dependent form of regulated cell death,^[^ ^6^ ^]^ which is triggered by a deadly accumulation of iron‐dependent membrane lipid peroxidation. When iron‐dependent lipoxygenase (LOXs) or reactive oxygen species (ROS) oxidize the polyunsaturated fatty acids (PUFAs) on phospholipid membranes to form toxic lipid peroxides (LPO), the cell membrane becomes unstable and permeable.^[^ ^7^ ^]^ Free iron accumulated in cells can promote lipid peroxidation via the Fenton reaction and generates ROS.^[^ ^8^, ^9^ ^]^ Upon various stresses, accumulation of lipid peroxidation can induce ferroptosis. To avoid ferroptosis, there are two main monitoring mechanisms to prevent it. First, glutathione (GSH) peroxidase 4 (GPX4) can reduce LPO to the accompanying phospholipid alcohol, thus GPX4, its upstream GSH synthesis, and cyst(e)ine inputs become the major mechanism for inhibiting ferroptosis.^[^ ^10^, ^11^ ^]^ The other is the production of metabolites exhibiting free radical trapping antioxidant (RTA) reactivity via enzymes such as GTP cyclohydrolase 1 (GCH1), dihydroorotate dehydrogenase (DHODH), and ferroptosis suppressor protein 1 (FSP1), thus terminating lipid peroxidation.^[^ ^12^, ^13^, ^14^ ^]^ Ferroptosis involves in various biological processes, such as increased tissue damage, cancer cell death, or cell death during tissue renewal.^[^ ^15^, ^16^ ^]^ In addition, ferroptosis can mediate the anticancer reactivity of several tumor suppressors, functioning as an innate tumor suppressive mechanism. Studies have demonstrated that suppression of ferroptosis and lipid peroxidation in colon tissue of high‐fat diet mice is accompanied by an increase in tumor number and hyperplasia.^[^ ^17^ ^]^ Recent studies demonstrated that cancer cells may use novel surveillance mechanisms independent of RTAs and GPX4 to evade ferroptosis, in which sex hormone signaling can suppress ferroptosis through membrane bound O‐acyltransferase domain containing 1/2 (MBOAT1/2)‐mediated phospholipid reprofiling in cancer cells.^[^ ^18^ ^]^ Thus, it would be particularly important to identify additional surveillance mechanisms of preventing ferroptosis, as it could help to develop new combination therapies.

Ether lipids as an essential class of phospholipids, including ether‐linked phosphatidylethanolamine (ePE) and ether‐linked phosphatidylcholine (ePC), are characterized by the attachment of an alkyl or alkenyl bond to the sn‐1 site of the glycerol backbone instead of an ester bond, and an abundance of PUFAs to the sn‐2 site.^[^ ^19^ ^]^ Common important metabolites of ePE and ePC include plasmenyl‐PE and platelet‐activating factor (PAF). Plasmenyl‐PE represents the most enriched alkenyl‐glycerophospholipid form, which is considered to be endogenous antioxidant.^[^ ^20^ ^]^ Conversely, PAF, which can be produced by a biologically inactive phospholipid named lyso‐PAF,^[^ ^21^ ^]^ is a lipid mediator that transmits cellular signals by binding with its receptor (PAF‐R), which activates pro‐thrombotic and pro‐inflammatory pathways under certain conditions thought to be associated with the onset and progression of inflammatory disease or atherosclerotic cardiovascular disease.^[^ ^22^ ^]^ The polyunsaturated ether lipids synthesized in peroxisomes induce ferroptosis by providing substrates for lipid peroxidation.^[^ ^23^, ^24^ ^]^ However, the molecular mechanisms of how changes in ether lipids induce ferroptosis in intestinal epithelial cells remain unclear.

*Selenoprotein I **(**SELENOI)* contains a rare selenium cysteine (Sec) amino acid in its active site. It is a unique membrane selenoprotein located exclusively in the lipid bilayer and can catalyze the intramembrane enzyme reaction itself, specifically catalyzing the biosynthesis of CDP ethanolamine to phosphatidylethanolamine (PE) (the last step on the Kennedy pathway).^[^ ^25^ ^]^ It has been shown that *SELENOI* is mainly responsible for the synthesis of PE that containing longer fatty acid chains and plasmenyl‐PE.^[^ ^26^ ^]^ Recently, two complicated cases of hereditary spastic paraplegia, a human autosomal recessive neurodegenerative disease associated with mutations in the *SELENOI* gene (named SPG81), confirmed that *SELENOI* plays important roles in human myelin formation and neural development by maintaining the balance of ether‐linked lipids.^[^ ^26^, ^27^ ^]^ Complete deletion of *Selenoi* resulted in early embryonic death in mice.^[^ ^28^ ^]^ Predictive studies of selenoprotein deletion tolerance in humans have shown that humans cannot tolerate deletion of even one of the alleles of *SELENOI*,^[^ ^29^ ^]^ and no pure mutations have been found in reported families of patients with *SELENOI* mutations in the above two cases, suggesting that *SELENOI* may be an essential selenoprotein for human health. In addition, the upregulated *SELENOI* expression was found in lung cancer tissue or melanoma cells.^[^ ^30^ ^]^ The importance of *SELENOI* in T cell activation and regulation of humoral immunity was also demonstrated.^[^ ^31^, ^32^ ^]^ However, whether *SELENOI* functions as a regulator of ferroptosis and involves in intestinal diseases (IBD or CRC) is currently unknown.

In this study, utilizing intestinal epithelium‐specific *Selenoi* conditional knockout (cKO) mice, we showed that *Selenoi* promotes intestinal epithelial regeneration during colitis and serves as an oncogenic factor in CAC. Mechanistically, *Selenoi* prevents ferroptosis by maintaining ether phospholipid homeostasis and downregulating phospholipase A2, group IIA (*Pla2g2a)* and group V (*Pla2g5)*, as well as arachidonate‐15‐lipoxygenase (*Alox15)*. Particularly, the regulation of *Selenoi* on ferroptosis is independent of GPX4. Overall, these findings indicated that *Selenoi* functions as a novel modulator for preventing ferroptosis.

## 2 Results

### 2.1 *Selenoi/SELENOI* is Expressed Mainly in Intestinal Epithelial Cells

**Figure 1:**
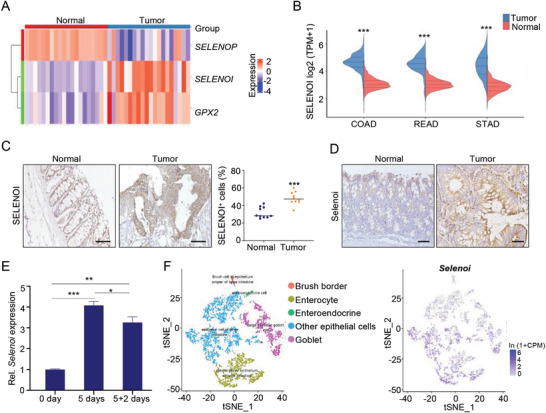
*Selenoi* is predominantly expressed in intestinal epithelial cells and elevated in DSS‐induced mouse colitis and AOM‐DSS‐induced mouse tumors. A) Heatmap of significantly changed selenoprotein genes in CRC patients from the GSE100179 RNA‐Seq dataset (normal = 20, tumor = 20). B) The *SELENOI* expression level in human tumors from TCGA and GTEx databases. COAD: colon adenocarcinoma, normal = 455, tumor = 820; READ: rectum adenocarcinoma, normal = 165, tumor = 789; STAD: stomach adenocarcinoma, normal = 375, tumor = 391. C) Immunohistochemical staining for *SELENOI* in CRC tissue and adjacent normal tissues. The percentage of *SELENOI* ^+ ^cells was quantified. Scale bar, 100 µm. *n *= 10. D) Immunohistochemical staining for *Selenoi* in adjacent normal tissues and AOM‐DSS colon tumors of mice. Scale bar: 50 µm. *n* = 5. E) qRT‐PCR analysis for *Selenoi* in colonic tissues from mice before DSS (0 day), 5 days after DSS treatment (5 days), and 2 days after removal of DSS (5 + 2 days), *n* = 4 for each time point. F) Single‐cell sequencing analysis of *Selenoi* expression levels in mouse large intestine epithelial from Tabula Muris database. The data are expressed by mean ± SD. *P < 0.05; **P < 0.01; ***P < 0.001.

Selenoproteins, which are the main form of selenium, play important roles both in physiological and pathological conditions. However, the functions of selenoproteins in colorectal cancer are unclear. The expression levels of selenoproteins in colorectal cancer were analyzed, and found that three selenoprotein genes were significantly altered in colorectal cancer (Table S1, Supporting Information). Selenoprotein P (*SELENOP)* was significantly downregulated, while glutathione peroxidase 2 (*GPX2)* and *SELENOI* were upregulated (**Figure** **1**A). TCGA and GEO datasets analysis showed that *SELENOI* was significantly upregulated in gastric, colonic, and rectal adenocarcinomas (Figure 1B). Compared to the adjacent normal tissues, *SELENOI* was significantly upregulated in CRC tissues (Figure 1C). In addition, in situ hybridization and immunohistochemical staining assays also revealed the upregulation of *Selenoi* in AOM‐DSS‐induced mouse colon tumors (Figure 1D; Figure S1A, Supporting Information), suggesting its potential role in tumorigenesis. Interestingly, we observed that DSS‐induced inflammation led to a significant upregulation of *Selenoi* expression in colitis, and after DSS removal, its expression gradually returned to basic levels (Figure 1E). To elucidate the potential role of *Selenoi* in intestinal homeostasis, we have investigated its expression pattern in the intestine, the single‐cell sequencing of mouse large intestinal tissues showed that *Selenoi* was extensively expressed in intestinal epithelial cells (Figure 1F). The above findings demonstrated that *Selenoi* is highly expressed in normal intestinal epithelial cells, and in colorectal tumors and colitis, suggesting its potential importance in intestinal homeostasis and diseases.

### 2.2 *Selenoi* is Essential for Intestinal Epithelial Regeneration in Mouse Model of Colitis

**Figure 2:**
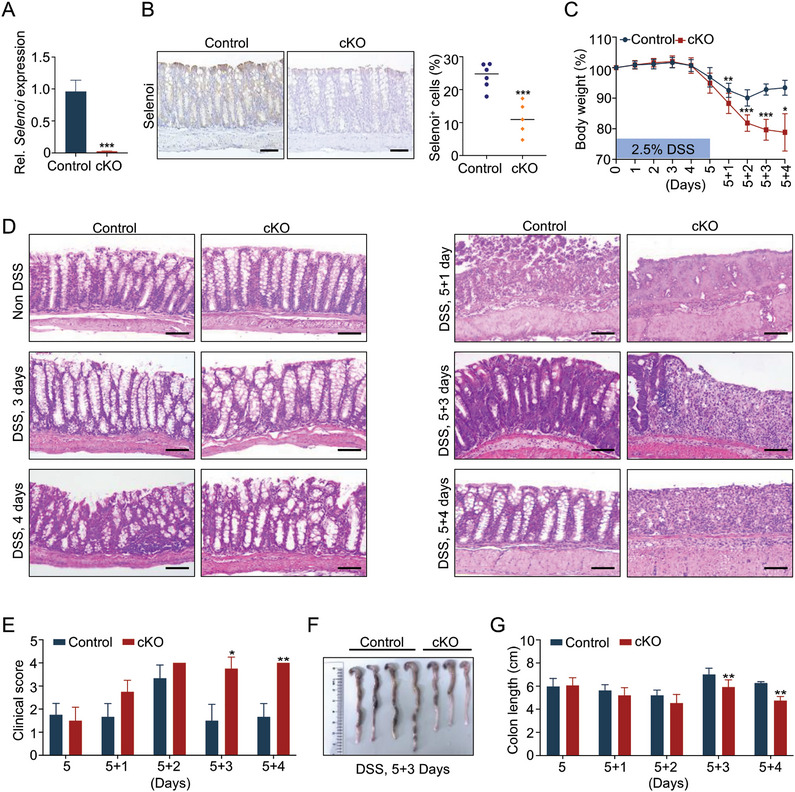
Deletion of *Selenoi* in epithelial cells impaired intestinal regeneration. A) qRT‐PCR analysis showed the expression levels of *Selenoi* in isolated colonic epithelial cells from control and cKO mice. *n* = 4. B) The *Selenoi* levels in colonic tissues of control and cKO mice detected by immunohistochemistry. Scale bar: 50 µm. *n* = 5. C) Quantification of body weight change in control (*n *= 23) and cKO (*n* = 25) mice at indicated time points after 2.5% DSS treatment and DSS removal. D) Histological images of colonic tissues from control and cKO mice at indicated time points of DSS induction. *n* = 3 at each timepoint. Scale bar: 50 µm. E) Quantification of the clinical scores in control (*n* = 23) and cKO (*n *= 25) mice at indicated time points of DSS treatment. F) Gross images of colons from control (*n* = 4) and cKO (*n* = 3) mice at 5 + 3 days after DSS treatment. G) Quantification of colon length from control (*n* = 23) and cKO (*n* = 25) mice at indicated timepoints of DSS treatment. The data are expressed by mean ± SD. *P < 0.05; **P < 0.01; ***P < 0.001. The cKO represents conditional deletion of *Selenoi* in intestinal epithelial cells.

To study the physiological function of *Selenoi* in the intestine, we generated *Selenoi* cKO mouse driven by Villin‐Cre, in which *Selenoi* was specifically deleted in the intestinal epithelium (Figure S1B–D, Supporting Information). In situ hybridization and qRT‐PCR assays showed that *Selenoi* was significantly deleted in intestinal epithelium from cKO mice at the mRNA levels (**Figure** **2**A; Figure S1E, Supporting Information). Immunohistochemical staining assays also showed that the protein levels of *Selenoi* were markedly reduced in intestinal epithelial cells from cKO mice (Figure 2B). *Selenoi* cKO mice were viable and fertile with no apparent gross phenotypes. No significant differences were found in the number of goblet cells, proliferative cells, and apoptotic cells within the crypt between control and cKO mice (Figure S2, Supporting Information), suggesting that *Selenoi* is dispensable for intestinal homeostasis under physiological conditions.

Next, the pathologic role of *Selenoi* in colitis was evaluated. Both control and cKO mice were given 2.5% DSS in drinking water for 5 days to induce colitis, followed by 4 days of recovery after DSS removal. The results showed that the body weight changes of control and cKO mice were almost similar during DSS treatment (Figure 2C). However, after the removal of the DSS, the body weights of cKO mice continued to decrease, whereas control mice appeared to recover toward the initial level (Figure 2C), indicating impaired intestinal epithelial regeneration in the absence of *Selenoi* expression. Correspondingly, compared to control mice, the colon tissue damage was not significantly aggravated in cKO mice during DSS administration, but the regeneration response was impaired after the removal of DSS (Figure 2D). In agreement with this finding, cKO mice had higher colon clinical scores and shorter colon lengths than the control mice 3 or 4 days after DSS removal (Figure 2E–G). The above findings suggest that *Selenoi* exerts an important role in promoting intestinal epithelial regeneration in colitis.

Uncontrollable prolonged inflammation promotes intestinal tumorigenesis, and together with ectopic overexpression of *Selenoi* in colorectal tumors, we assume that *Selenoi* might aggravate intestinal tumorigenesis. Thus, we used the AOM‐DSS‐induced colorectal tumor mouse model to explore this possibility of *Selenoi*. The results showed that the absence of *Selenoi* leading to a significant decrease in the colonic number of polyps and polyp load (Figure S3A–D, Supporting Information). The areas of the hyperplastic lesion were significantly reduced in colon tumors from cKO mice (Figure S3E, Supporting Information), relative to control. Consistently, the percentage of *Selenoi* ^+^ cells or Ki67^+^ cells was markedly decreased in cKO mice (Figure S3F,G, Supporting Information). Tumor xenograft assays also showed that *SELENOI* deficiency significantly inhibited tumor growth (Figure S3H,I, Supporting Information). These findings suggested that intestinal epithelial cell‐derived *Selenoi* plays an oncogenic role in colorectal tumorigenesis.

### 2.3 Epithelial *Selenoi* Suppresses the Ferroptosis Pathway by Balancing Ether Lipids

**Figure 3:**
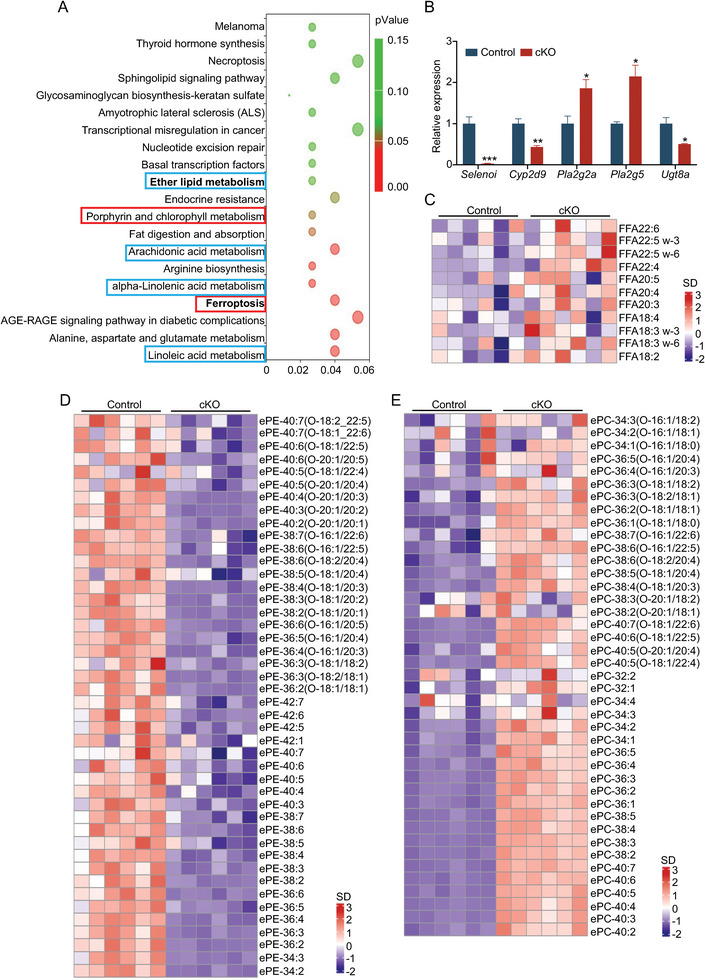
Deletion of *Selenoi* in epithelial cells disrupts ether lipid homeostasis. A) KEGG enrichment analysis of differentially expressed genes in isolated colonic epithelial cells of cKO mice. Red boxes represent pathways associated with ferroptosis and blue boxes represent pathways associated with ether lipid metabolism. B) qRT‐PCR analysis validates altered ether lipid metabolism‐related genes in isolated colonic epithelial cells from control and cKO mice. *n *= 4. C) Heatmap of PUFAs in colonic tissues of control and cKO mice. *n* = 6. D,E) Heatmap of ePE (D) and ePC (E) in colonic tissues of control and cKO mice. *n* = 6. The data are expressed by mean ± SD. *P < 0.05; **P < 0.01; ***P < 0.001. cKO represents conditional deletion of *Selenoi* in intestinal epithelial cells.

To gain insight into the molecular mechanism underlying the regulatory effect of *Selenoi* on epithelial regeneration and tumor growth, the genome‐wide transcriptome analysis was performed on colonic epithelial cells from control and cKO mice. We identified 188 upregulated genes and 221 downregulated genes in cKO mice (Figure S4A, Supporting Information). KEGG analysis revealed that pathways most enriched among downregulated genes include ferroptosis and ether lipids metabolism (including PUFAs metabolism) (**Figure** **3**A). qRT‐PCR assays validate this alteration (Figure 3B). It suggested that *Selenoi* deficiency might be involved in promoting ferroptosis. Consistent with this idea, we also found a significant upregulation of PUFAs whose overaccumulation contributes to lipid peroxidation (Figure 3C). Furthermore, we performed lipidomics analysis and found that *Selenoi* deficiency directly caused a remarkable reduction of ePE (Figure 3D), since *SELENOI* functions as ethanolamine phosphotransferase 1. The reduction of ePE resulted in excessive accumulation of ePC (Figure 3E) since they share a common substrate 1‐O‐alkyl‐2‐acyl‐sn‐glycerol (DG‐O). It has been reported that the imbalance of ePE and ePC can induce ferroptosis susceptibility.^[^ ^24^ ^]^ In addition, *Selenoi* deficiency resulted in mild changes in phosphatidylethanolamine and phosphatidylcholine (Figure S4B–F, Supporting Information). Taken together, the above findings suggest that loss of *Selenoi* mainly causes the remodeling of ether lipids that activate the ferroptosis pathway, consequently resulting in impaired intestinal epithelial regeneration and compromised tumor growth.

**Figure 4:**
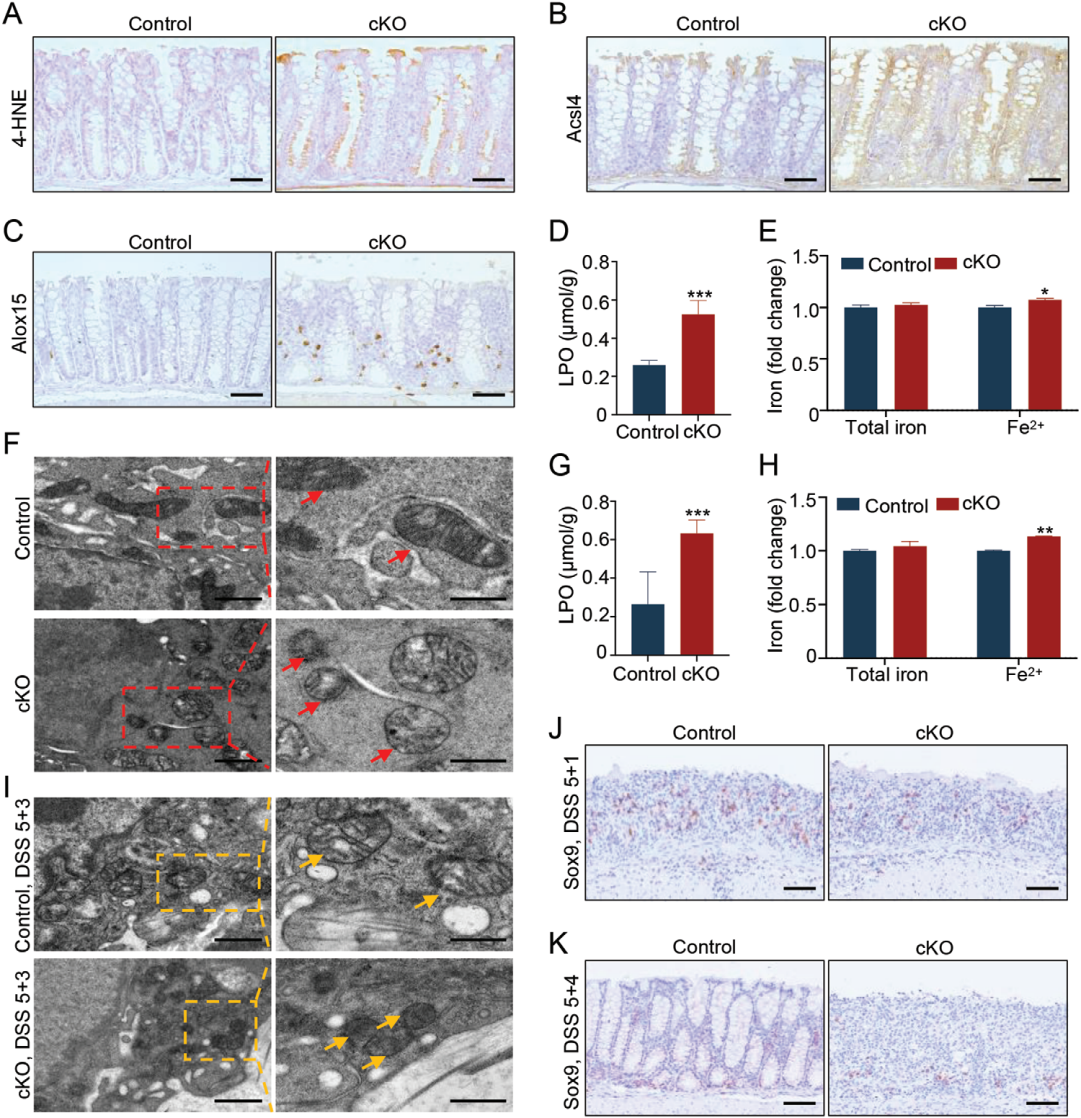
*Selenoi* deficiency in epithelial cells promotes ferroptosis. A–C) Immunohistochemical staining for 4‐HNE (A), Acsl4 (B), and Alox15 (C) in colonic tissues of control and cKO mice. Scale bar = 50 µm. *n* = 3. D) Quantitative analysis of LPO, an indicator of lipid peroxidation, in colonic tissues of control and cKO mice; *n* = 4. E) Quantitative analysis of total iron and Fe^2+^ levels in colonic tissues of 4 control and 4 cKO mice. F) Representative TEM images of colonic tissues from control and cKO mice. High‐magnification of mitochondrial ultrastructure is on the right. Red arrows point to mitochondria. Scale bar: 1 µm (left panel) and 500 nm (right panel). G) Variation of LPO content in colonic tissues from control and cKO mice on 5 + 3 days after DSS treatment. *n *= 4. H) Quantification of total iron and Fe^2+^ levels in colonic tissues from control and cKO mice on 5 + 4 days after DSS treatment. *n* = 4. I) Representative TEM images of colonic tissues from control and cKO mice on 5 + 3 days after DSS treatment. High‐magnification of mitochondrial ultrastructure is on the right. Yellow arrows point to mitochondria. Scale bar: 1 µm (left panel) and 500 nm (right panel). J,K) Immunohistochemical staining for Sox9 in colonic tissues of control and cKO mice on 5 + 1 days (J) and 5 + 4 days (K) after DSS treatment. Scale bar: 50 µm. The data are expressed by mean ± SD. *P < 0.05; **P < 0.01; ***P < 0.001. cKO represents conditional deletion of *Selenoi* in intestinal epithelial cells.

Furthermore, the metabolic enzymes of arachidonic acid (AA) including acyl‐CoA synthetase long‐chain family member 4 (ACSL4), ALOX15, and cyclooxygenase 2 (COX2) are often used as markers of ferroptosis, as they can produce different lipid metabolites to induce ferroptosis.^[^ ^16^ ^]^ To investigate the role of *Selenoi* in ferroptosis, changes in ferroptosis‐related genes enriched for KEGG were first confirmed by qRT‐PCR assays (Figure S5A, Supporting Information). Then we examined the levels of the ferroptosis markers, as well as LPO. The immunohistochemical assay showed that 4‐HNE, Acsl4, and Alox15 were significantly upregulated in colonic epithelium upon *Selenoi* deletion (**Figure** **4**A–C). Consistently, we also found upregulation of 4‐HNE, Acsl4, and Alox15 in *SELENOI*‐deficient HCT116 cells (Figure S5B, Supporting Information). It is well known that lethal accumulation of iron‐dependent lipid peroxidation leads to ferroptosis.^[^ ^16^ ^]^ Compared with control mice, the contents of LPO and ferrous iron (Fe^2+^) are significantly elevated in the colonic tissues of cKO mice (Figure 4D,E). Furthermore, transmission electron microscopy (TEM) assay showed that *Selenoi* deficiency led to dysmorphic mitochondria and small mitochondria with increased membrane density, mimicking the mitochondrial phenotypes of ferroptotic cell death (Figure 4F). In addition, the upregulation of the ferroptosis markers was also observed both in cKO colonic tissues during the regenerative stage and in AOM‐DSS‐induced colon tumors from cKO mice (Figure S5C–F, Supporting Information). The LPO and Fe^2+^ levels were significantly increased in the colonic tissues of cKO mice during the regenerative stage (Figure 4G,H). The mitochondrial structural damage was also observed in the colonic tissues of cKO mice 3 days after DSS removal (Figure 4I). Interestingly, immunohistochemical staining showed that the abundance of sex‐determining region y‐box 9^+^ (Sox9^+^) stem/progenitor cells was much lower in colonic tissues of cKO mice 1 or 4 days after DSS removal, compared with control mice (Figure 4J,K), which is most likely caused by ferroptosis. Taken together, these findings demonstrated that the deletion of *Selenoi* enhanced promotes the occurrence of ferroptosis in intestinal epithelial cells, especially in Sox9^+^ Stem/progenitor cells. It suggests that *Selenoi* plays an essential part in enhancing epithelial regeneration during colitis by suppressing ferroptosis.

### 2.4 Loss of *SELENOI* Increases Susceptibility of Colorectal Cancer Cells to Ferroptosis

**Figure 5:**
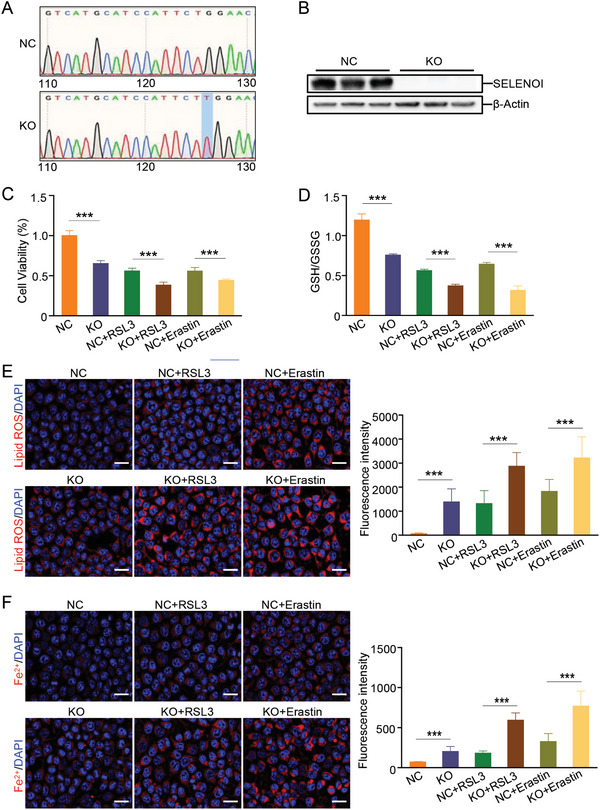
*SELENOI* deficiency increases ferroptosis susceptibility in vitro. A) Sanger sequencing of normal HCT 116 cells (NC) and *SELENOI* deficient (KO) HCT 116 cells. B) Western blotting for *SELENOI* in NC and KO cells. *β*‐Actin was used as a loading control. C) Cell viability analysis of NC and KO cells upon RSL3 (3 µm for 24 h) or Erastin (20 µm for 24 h) treatment. *n* = 5. D) GSSG/GSH ratio of NC and KO cells upon RSL3 or Erastin treatment. *n* = 5. E,F) Lipid ROS staining (E) and FerroOrange staining (F) of NC and KO cells upon RSL3 or Erastin treatment. The fluorescence intensity was quantified. Scale bar: 25 µm. *n* = 5. The data are expressed by mean ± SD. ***P < 0.001.

To further solidify the importance of *SELENOI* in ferroptosis, we generated a *SELENOI* deficient (KO) HCT116 cell line using the CRISPR/Cas9 system (**Figure** **5**A,B). We found that the viability of SELENOI KO HCT116 cells was significantly reduced, accompanying by a decrease of the GSH/GSSG ratio. The decrease in cell viability and the GSH/GSSG ratio became more pronounced in SELENOI KO HCT116 cells upon treatments with ferroptosis inducers RSL3 or Erastin (Figure 5C,D). Immunofluorescence staining showed that the intensity of lipid ROS and Fe^2+^, which are markers of ferroptosis, was remarkably increased in SELENOI KO cells, and became more pronounced upon treatments of ferroptosis inducers (Figure 5E,F). Similar findings were observed in siRNA‐mediated *SELENOI* knockdown cells. The viability of *SELENOI* knockdown cells significantly decreased with increasing doses of RSL3 (Figure S6A,B, Supporting Information). Knockdown of SELENOI significantly increased ROS production and mitochondrial damage, and they become more severe upon RSL3 treatment (Figure S6C,D, Supporting Information). These findings indicated that loss of *SELENOI* enhances ferroptosis susceptibility of colorectal cancer cells.

### 2.5 SELENOI Suppresses Ferroptosis in a GPX4‐Independent Manner

**Figure 6:**
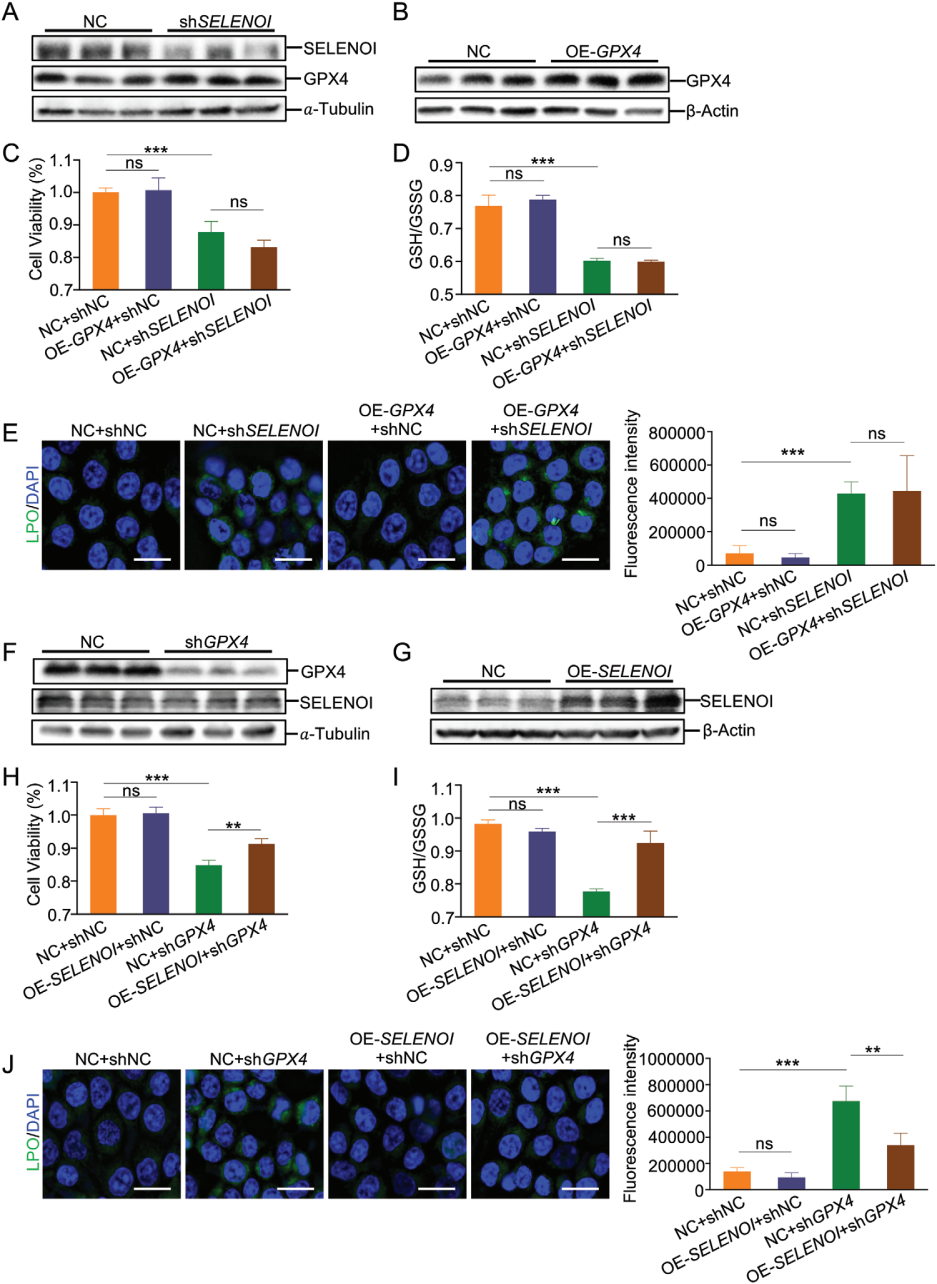
*SELENOI* overexpression significantly alleviates GPX4 inhibition‐induced ferroptosis in vitro. A) Western blotting for *SELENOI* and GPX4 in HCT116 cells infected with shNC or sh*SELENOI*. *α*‐Tubulin was used as the loading control. B) Western blotting for GPX4 in NC and GPX4 overexpression (OE‐GPX4) HCT116 cells. *β*‐Actin was used as the loading control. C) Cell viability analysis of NC and GPX4 overexpression HCT116 cells infected with shNC or sh*SELENOI*. *n *= 5. D) GSSG/GSH ratio of NC and GPX4 overexpression HCT116 cells infected with shNC or sh*SELENOI*. *n* = 5. E) Liperfluo staining of NC and GPX4 overexpression HCT116 cells infected with shNC or sh*SELENOI*. The fluorescence intensity was quantified. Scale bar: 25 µm. *n* = 5. F) Western blotting for GPX4 and *SELENOI* in HCT116 cells infected with shNC or shGPX4. *α*‐Tubulin was used as the loading control. G) Western blotting for *SELENOI* in NC and *SELENOI* overexpression (OE‐*SELENOI*) HCT116 cells. *β*‐Actin was used as the loading control. H) Cell viability analysis of NC and *SELENOI* overexpression HCT116 cells infected with shNC or shGPX4. *n* = 5. I) GSSG/GSH ratio of NC and *SELENOI* overexpression HCT116 cells infected with shNC or shGPX4. *n* = 5. J) Liperfluo staining of NC and *SELENOI* overexpression HCT116 cells infected with shNC or shGPX4. The fluorescence intensity was quantified. Scale bar: 25 µm. *n* = 4. The data are expressed by mean ± SD. **P < 0.01; ***P < 0.001.

It has been reported that GPX4 plays an integral role in the inhibition of ferroptosis.^[^ ^16^, ^33^, ^34^ ^]^ To investigate whether GPX4 is involved in the process of *SELENOI* deficiency‐induced ferroptosis, we first examined GPX4 expression in *SELENOI* knockdown cells or in colonic tissues from *Selenoi* cKO mice. Interestingly, GPX4 showed an increasing trend in *SELENOI* knockdown cells, and it is markedly elevated in *Selenoi* deficient colonic tissues (Figure S6E,F, Supporting Information). Next, we overexpressed GPX4 in *SELENOI* knockdown cells (**Figure** **6**A,B). Surprisingly, there was no significant change in cell viability values and the GSH/CSSG ratio in *SELENOI* knockdown cells upon GPX4 overexpression (Figure 6C,D). Its overexpression did not inhibit the generation of LPO (Figure 6E). It means that GPX4 cannot rescue the *SELENOI* knockdown‐induced ferroptosis. In contrast, we overexpressed *SELENOI* in *GPX4* knockdown cells (Figure 6F,G), and found that *SELENOI* overexpression partially alleviated the decreased cell viability and the increased oxidative stress (Figure 6H,I). In addition, *SELENOI* overexpression resulted in a significant decrease in LPO fluorescence intensity in *GPX4* knockdown cells (Figure 6J). Taken together, these findings suggested that *SELENOI* suppresses ferroptosis independent of GPX4.

### 2.6 *SELENOI* Deficiency‐Mediated Ferroptosis can be Rescued by Suppression of *PLA2G2A*, *PLA2G5* or *ALOX15*

**Figure 7:**
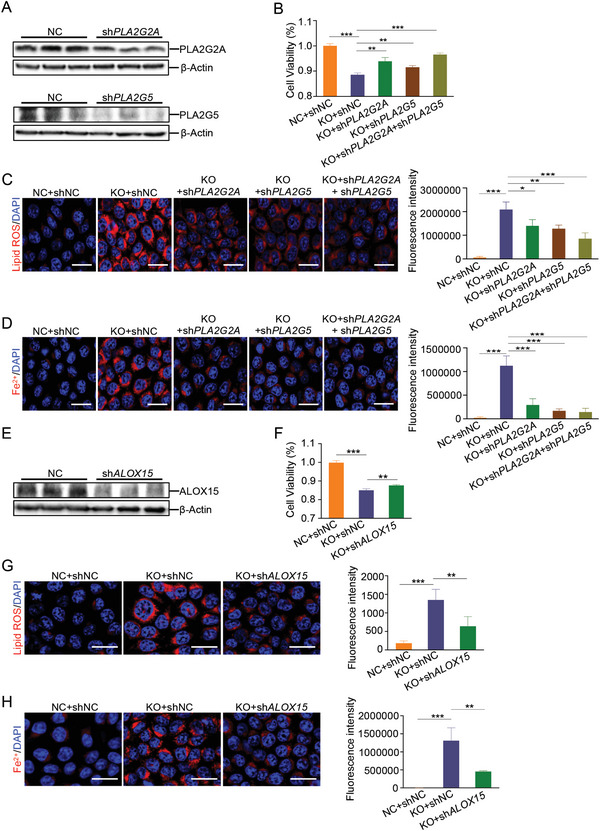
*SELENOI*‐mediated ferroptosis can be rescued by suppression of PLA2G2A, PLA2G5, or ALOX15. A) Western blotting for PLA2G2A in HCT116 cells infected with shNC or shPLA2G2A, and PLA2G5 in HCT116 cells infected with shNC or shPLA2G5. *β*‐Actin was used as the loading control. B) Cell viability analysis of normal HCT 116 cells (NC) and *SELENOI* deficient (KO) HCT116 cells infected with shNC, shPLA2G2A, or shPLA2G5. *n* = 5. C,D) Lipid ROS staining (C) and FerroOrange staining (D) of NC and KO cells infected with shNC, shPLA2G2A or shPLA2G5. The fluorescence intensity was quantified. Scale bar: 25 µm. *n* = 5. E) Western blotting for ALOX15 in HCT116 cells infected with shNC or shALOX15. *β‐*Actin was used as the loading control. F) Cell viability analysis of NC and KO cells infected with shNC or shALOX15. *n* = 5. G, H) Lipid ROS staining (G) and FerroOrange staining (H) of NC and KO cells infected with shNC or shALOX15. The fluorescence intensity was quantified. Scale bar: 25 µm. *n* = 5. The data are expressed by mean ± SD. **P < 0.01; ***P < 0.001.

To further investigate the molecular mechanism underlying *SELENOI* deficiency‐mediated ferroptosis, we screened the differentially expressed genes in the transcriptomic data. The significant upregulation of *Pla2g2a*, *Pla2g5*, and *Alox15* was observed in colonic epithelial cells from *Selenoi* cKO mice (Figure 3B; Figure S5A, Supporting Information). Pla2g2a and Pla2g5 belong to the phospholipase A2 enzyme family and catalyze the hydrolysis of the sn‐2 acyl bonds of glycerophospholipids, producing lysophospholipids and free fatty acids, mostly PUFAs,^[^ ^35^ ^]^ whereas Alox15 mainly oxidizes PUFAs, particularly omega‐6 and −3 fatty acids, to generate abundant of bioactive lipid metabolites that contribute to ferroptosis.^[^ ^36^ ^]^ In vitro assay also showed that *PLA2G2A*, *PLA2G5* or ALOX15 is upregulated in *SELENOI* knockdown cells (Figures S4G,S5B, Supporting Information), suggesting a direct impact of *SELENOI* on their expression. Consistently, *SELENOI* was significantly upregulated in human colorectal cancer tissues (Figure 1C), while PLA2G2A, PLA2G5, and ALOX15 were significantly downregulated (Figure S7, Supporting Information). To test whether PLA2G2A, PLA2G5, or ALOX15 upregulation mediates the *SELENOI* deficiency‐induced ferroptosis, we knockdown them in *SELENOI* deficient cells. Loss of *SELENOI* led to a decrease in cell viability and an increase in lipid ROS and Fe^2+^ while these effects were reversed by suppressing PLA2G2A, PLA2G5, or ALOX15 expression (**Figure** **7**A–H). Overall, the data indicated that PLA2G2A, PLA2G5, and ALOX15 function as downstream effectors of *SELENOI* in regulating ferroptosis.

### 2.7 Supplement of Selenomethionine Alleviates *Selenoi* Deficiency‐Mediated Impairment of Intestinal Epithelial Regeneration

**Figure 8:**
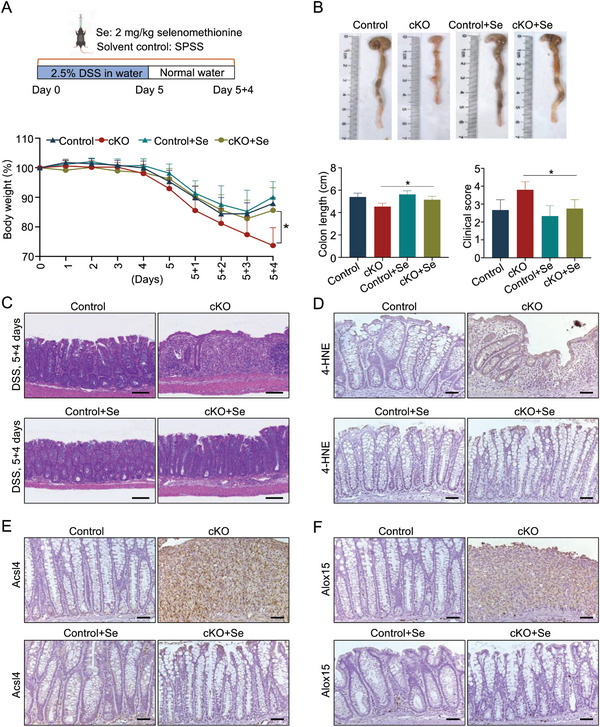
Selenium alleviates the impaired intestinal epithelial regeneration induced by *Selenoi* deficiency in the DSS colitis model. A) Schematic diagram of selenomethionine induction (upper panel) and quantification of body weight change in control (*n* = 6) and cKO (*n* = 6) mice treated with selenomethionine (lower panel). B) Gross images of colons (upper panel), quantification of colon length (left panel), and the clinical scores (right panel) from control and cKO mice at 5 + 4 days after selenomethionine treatment. *n* = 6. C) Histological images of colonic tissues from control and cKO mice at 5 + 4 days after selenomethionine treatment. Scale bar: 100 µm. *n* = 6. D–F) Immunohistochemistry for 4‐HNE (D), Acsl4 (E), and Alox15 (F) in colonic tissues from control and cKO mice at 5 + 4 days after selenomethionine treatment. Scale bar: 50 µm. *n* = 3. SPSS represents stroke‐physiological saline solution; Control+Se represents control mice treated with selenomethionine; cKO+Se represents cKO mice treated with selenomethionine. The data are expressed by mean ± SD. *P < 0.05.

The above findings indicated that *Selenoi* deficiency impairs intestinal epithelial regeneration during colitis due to lipid peroxidation‐induced ferroptosis. It has been well known that selenomethionine exerts antioxidant effects.^[^ ^37^, ^38^, ^39^ ^]^ We thus test whether selenomethionine can alleviate *Selenoi* deficiency‐induced lipid peroxidation and defective intestinal regeneration. We induced mouse colitis using DSS and then administered additional selenomethionine (2 mg kg^−1^) to mice. The addition of selenomethionine significantly rescued the severe spontaneous colitis phenotypes of cKO mice during the regenerative stage, as evidenced by slower loss of body weight, lower clinical scores, increased colon length, and enhanced epithelial regeneration 4 days after DSS removal (**Figure** **8**A–C). Selenomethionine treatment decreased the levels of 4‐HNE, Acsl4, and Alox15 in the colon of cKO mice during intestinal epithelial regeneration (Figure 8D–F). The data indicated that selenomethionine can alleviate lipid peroxidation‐mediated ferroptosis caused by *Selenoi* deficiency, and thereby improved intestinal regeneration.

## 3 Discussion

In the study, we demonstrated that *Selenoi* promotes intestinal epithelial regeneration by maintaining ether lipids homeostasis to inhibit the ferroptosis signaling pathway, revealing a new feature of *Selenoi* in gut diseases. Consistent with this finding, other studies have suggested that ferroptosis was associated with intestinal epithelial cell mortality in ulcerative colitis and intestinal epithelial cytotoxicity in IR mice.^[^ ^40^, ^41^ ^]^ It has been reported that endoscopic manifestations and clinical symptoms of ulcerative colitis (UC) patients were significantly improved and ROS in colonic tissues were significantly reduced after administration of iron chelators.^[^ ^42^, ^43^ ^]^ Moreover, dietary supplementation with high iron exacerbated UC symptoms in a mouse model or two patients.^[^ ^44^, ^45^ ^]^ On the other hand, reprogramming and sufficient reorganization of lipid metabolism seems to be the integral event resulting in an effective inflammatory response.^[^ ^46^ ^]^ A diet enriched in omega‐6 PUFAs such as arachidonic acid, increases the incidence of IBD.^[^ ^47^ ^]^ However, the alkenyl ether groups of ether lipids prevent colitis and inhibit ferroptosis.^[^ ^48^ ^]^ Thus, it appears that preventing ferroptosis could be a valid strategy for IBD intervention.

We found that *Selenoi* deficiency directly caused a significant decrease in ePE and excessive accumulation of ePC. This ether lipid imbalance provided more PUFAs for lipid peroxidation, ultimately leading to ferroptosis. Indeed, the synthesis of polyunsaturated ether phospholipids promotes ferroptosis by providing substrates for lipid peroxidation.^[^ ^24^ ^]^ Similarly, a recent study revealed a novel surveillance mechanism to suppress ferroptosis, in which sex hormone signaling can suppress ferroptosis through MBOAT1/2‐mediated increases in cellular PE‐monounsaturated fatty acids and corresponding decreases in cellular PE‐polyunsaturated fatty acid, independent of GPX4 and FSP1.^[^ ^18^ ^]^ It turns out that the imbalance of polyunsaturated ether phospholipids is a major cause of ferroptosis. Furthermore, the fact that the expression levels of GPX4 increased when ferroptosis occurred caused by *Selenoi* deficiency, and GPX4 overexpression cannot rescue the ferroptosis phenotypes in *SELENOI*‐knockdown cells, supporting the notion that *SELENOI* prevents ferroptosis independent of GPX4. As is well known, selenium is an essential element that contributes to maintain redox homeostasis^[^ ^38^ ^]^ via selenoprotein‐mediated cellular reactive oxygen species scavenging.^[^ ^37^, ^39^, ^49^ ^]^ Consistently, we found that selenium supplementation via selenomethionine alleviates lipid peroxidation‐mediated impairment of intestinal epithelial regeneration caused by *Selenoi* deficiency. Taken together, we hypothesize that the antioxidant effects of selenomethionine supplementation mediated intestinal epithelial regeneration are not due to the upregulation of GPX4, but potentially due to the upregulation of other selenoproteins in this study.

Interestingly, here we found that *Selenoi* deficiency suppressed colon tumor growth, suggesting its oncogenic role. In agreement with its oncogenic function, it has been reported that an eicosanoid metabolite named as 12,13‐epoxyoctadecenoic acid (EpOME), produced from linoleic acid by CYP monooxygenase, enhanced the JNK phosphorylation and cytokine production and contributed to the development of AOM‐DSS‐induced colon tumors in vivo.^[^ ^50^ ^]^ Indeed, we observed that conditional deletion of *Selenoi* in intestinal epithelial cells resulted in a reduction of *Cyp2d9*, a CYP monooxygenase for the synthesis of 12,13‐EpOME. Thus, it is reasonable to assume that the reduction of 12,13‐EpOME may also account for compromised tumor growth. In addition, SOX9, a key regulatory factor for intestine development, is identified as a marker for intestinal stem/progenitor cells, and it is also associated with tumorigenesis and cancer progression.^[^ ^51^ ^]^ In the present study, we found that *Selenoi* deficiency resulted in a reduction of Sox9^+^ stem/progenitor cells in colitis and a subsequent decrease in tumors from the AOM‐DSS colorectal cancer mouse model. Consistently, it has been reported that elevated Sox9 contributed to the development of colorectal cancer.^[^ ^51^ ^]^ Thus, it replies that *Selenoi* plays its oncogenic role in CRC via multiple ways. In fact, the development of intestinal tumors was also regulated by other selenoproteins. Deletion of *SELENOP* in intestinal epithelial cells may promote cell transformation and tumorigenesis by increasing oxidative stress and genomic instability,^[^ ^52^ ^]^ Selenoprotein H was associated with the control of cell cycle and proliferation of the human colorectal cancer cells,^[^ ^53^ ^]^ and Selenoprotein F may be a potential determinant of the types of colorectal tumor development.^[^ ^54^ ^]^ Glutathione peroxidase 3 showed potent tumorigenic effects in colitis‐associated cancer by eliminating ROS inhibitory effects.^[^ ^55^ ^]^ In the human colorectal cancer cell line, dysregulation of ether lipids increased the sensitivity to ferroptosis. Previous reports showed that the ferroptosis sensitivity in gastric cancer was determined by the polyunsaturated fatty acid biosynthesis pathway.^[^ ^56^ ^]^ Overall, it turns out that selenoproteins might have distinct functions in the development of CRC.

In summary, we demonstrated that intestinal epithelial *Selenoi* plays critical roles in intestinal epithelial regeneration and tumorigenesis by regulating ether lipids homeostasis and suppressing ferroptosis. When *Selenoi* was deficient, ePC increased excessively, promotes lipid peroxidation that induces ferroptosis in intestinal epithelial cells, which consequently impairs intestinal regeneration from colitis and compromises colon tumorigenesis. This finding offers novel perspectives for understanding the role of *Selenoi* in gut diseases and provides a scientific basis for new therapeutic strategies for colitis and colorectal cancer.

## 4 Experimental Section

### Animals

All mouse experimental procedures were performed in strict accordance with the guiding rules of the Institutional Animal Care and Use Committee of the China Agricultural University (approval number: AW71602202‐5‐32). *Selenoi*‐floxed mice were generated at Cyagen Biosciences (no: TOS171114ZQ1), and exons 2–3 (1558 bp) of *Selenoi* were targeted with flanking LoxP sites, resulting in two LoxP loci. *Villin‐Cre* mice were obtained from Zhengquan Yu's lab at China Agricultural University (stock no. T000142 from Jackson Laboratories).

### Patients and Clinical Specimens

Human CRC tissue microarray was obtained from Henan Cancer Hospital. Microarray contained 10 pairs of benign colorectal tissues and cancer tissues. Detailed information on the microarray is summarized in Table S2 (Supporting Information). All patients were from the Henan Cancer Hospital. The use of clinical samples and review of all pertinent patient records was approved by the Ethical Committee and Institutional Review Board of Henan Cancer Hospital (No. 2012‐KY‐0121‐001) in compliance with ethical standards and patient confidentiality, and informed consent was obtained from all patients.

### RNAscope In Situ Hybrid Dization Analysis

RNAscope in situ hybridization detection was carried out as previously described.^[^ ^1^ ^]^ Tissue sample sections were assayed with the RNAscope 2.5 HD Reagent Kit‐Red (cat. #322 350; ACDBio) according to the manufacturer's instructions. Briefly, sections were treated with pretreatment reagents to expose the target RNA. Gene‐specific probes were hybridized to target RNA. The probes were hybridized to a series of signal‐amplifying molecules, which further enhanced the signal, Fast Red substrate was added to detect target RNA. The sections were then counterstained in hematoxylin and dehydrated, finally mounted on a Vector Lab Vectamount (cat. #321 584; ACDBio). The target RNA was observed with a standard bright‐field microscope. DapB (cat. #310 043; ACDBio), Mm‐*Selenoi* (cat. #525 371; ACDBio) and Mm‐Ppib (cat. #313 911; ACDBio) were used in this experiment.

### DSS Treatment

Mice were fed 2.5% w/v DSS (MP Biochemicals, molecular weight 36 000–50 000) in drinking water for 5 days to induce acute colitis. Then the DSS was taken out of service and restored for a period of 4 days with a normal water supply. The severity of colitis was scored daily on the basis of body weight, diarrhea, and/or bloody stools. Mice were executed at different DSS treatment time points to obtain colon samples. Clinical scores were measured and modified as previously reported.^[^ ^57^ ^]^

### Selenomethionine Treatment

Ten‐week‐old control and cKO mice were fed 2.5% w/v DSS in drinking water for 5 days. Then, DSS was withdrawn for an additional 4 days. Selenium‐*L*‐methionine (S3132‐100MG, Sigma‐Aldrich, 2 mg kg^−1^) or stroke‐physiological saline solution was gavaged every day for the entire process after DSS induction.

### AOM‐DSS Mouse Model

The AOM–DSS mouse model was created as mentioned previously with some modifications.^[^ ^52^ ^]^ Briefly, 8‐week‐old control and *Selenoi* cKO mice were injected intraperitoneally with AOM (A5486‐25MG, Sigma‐Aldrich) at a concentration of 10 mg kg^−1^ body weight. Seven days after the AOM injection, mice were subjected to a DSS cycle: the mice were fed 2.5% w/v DSS (molecular weight: 36 000–50 000, MP Biomedicals) for 5 days and then normal water for 16 days. The processes were repeated three times. Tissue was collected from the distal colon and tumor number and volume were assessed. The number of polyps refers to the total number of polyps in the mouse, and the polyp load refers to the sum of the diameters of all polyps in the mouse.

### Xenograft Tumor

Five‐week‐old male nude mice were obtained (BALB/c) from SPF Biotechnology Co., Ltd. and maintained in specific pathogen‐free conditions. Mice were randomly grouped and subcutaneously injected with 5 × 10^6^ HCT116 cells in a total volume of 100 µL PBS. Mice were sacrificed two weeks after transplantation, tumor length and width were measured. The tumor volume was calculated as 0.5 × length × width^2^.

### qRT–PCR Analysis

Total RNA was obtained from cell lines and mouse colorectal tissues using TRIzol reagent (15596026CN, Life Technologies). Hieff qPCR SYBR Green Master Mix (11203ES08, Yeasen) and an ABI 7500 Real‐Time PCR System (Applied Biosystems, USA) were used to detect mRNA levels. Relative expression was calculated according to the 2^−▵▵CT^ method and, *Gapdh* was used as the internal control.

### Histology and Immunostaining

For histological analysis, colorectal tissues from control or cKO mice were fixed in 10% formalin, embedded in paraffin, and 4 µm sections were made. The sections were treated with xylene to remove paraffin and different concentrations of ethanol. They were later stained with hematoxylin (Sigma‐Aldrich) and eosin (Sigma‐Aldrich). After dehydration by serially diluted ethanol, the sections were mounted on coverslips in a neutral gel loading medium.

For immunostaining, the sections were microwaved in 0.01 m citrate buffer (pH 6.0) for antigen repair. Sections were cooled naturally, rinsed in ddH_2_O, soaked in 3% H_2_O_2_, rinsed in 1 × PBS, and then blocked with a blocking solution (TBS‐T containing 10% goat serum) at RT for 1 h. Sections were incubated with primary antibody overnight at 4 °C, rinsed with PBS, and incubated with goat anti‐mouse or anti‐rabbit IgG (H + L) at RT for 1 h. After rinsing with PBS, the sections were stained with DAPI for 5 min, and then covered with anti‐quenching reagent. The antibodies used are as follows: anti‐*Selenoi* (1:200, ab194554, Abcam), anti‐Pla2g2a (1/200, ab23705, Abcam), anti‐Pla2g5 (1:50, sc‐393606, Santa Cruz), anti‐Alox15 (1:4000, ab244205, Abcam), anti‐4HNE (1:200, ab46545, Abcam), anti‐Gpx4 (1:400, ab125066, Abcam), anti‐Facl4 (1:200, ab155282, Abcam), anti‐Ki67 (1:200, ab15580, Abcam), anti‐*β*‐catenin (1:200, sc‐7963, Santa Cruz), anti‐Mucin2 (1:2000, ab272692, Abcam), anti‐Sox9 (1:600, 82 630, CST), anti‐Cleaved‐caspase3 (1:2000, 9664, CST), and anti‐Phospho‐stat3 (1:400, 9145, CST).

### Iron Assay

Intracellular ferrous (Fe^2+^) and total iron levels were quantified using an iron assay kit (MAK025, Sigma‐Aldrich). Colorectal tissues (10 mg) from control and cKO mice were rapidly homogenized on ice with a fivefold volume of iron assay buffer and centrifuged (13 000 × g, 10 min) at 4 °C to remove insoluble material. An iron probe (100 µL) was added to each sample, mixed, and incubated at 25 °C for 60 min, during which time the plate was placed in the dark. The absorbance at 593 nm was measured immediately using a microplate reader.

### Transmission Electron Microscopy (TEM)

The colonic tissues were fixed with 2.5% glutaraldehyde in the dark at 4 °C for 12 h immediately after isolation. ultrathin sections were prepared and examined under transmission electron microscopy.

### Lipid Peroxide (LPO) Assay

Harvested colonic tissues were homogenized in physiological saline and centrifuged at 2500 rpm min^−1^ for 10 min. Then, the amount of LPO in colonic tissue was measured with a lipid peroxidation assay kit (A106‐1‐3, Nanjing Jiancheng Bioengineering Institute) according to the manufacturer's instructions.

### Lipidomics Assay

Lipids were extracted from ≈30 mg of tissue in the frozen state using a modified Bligh and Dyer method.^[^ ^58^ ^]^ Tissue homogenization was performed with 750 µL of chloroform: methanol: MilliQ water (3:6:1) (v/v/v). The homogenates were incubated at 4 °C and 1500 rpm for 1 h. After incubation, 350 µL of deionized water and 250 µL of chloroform were added for phase separation. The samples were then centrifuged and the lower organic phase containing lipids was extracted into a new EP tube. The extraction was repeated once with 450 µL of chloroform in the remaining aqueous phase, and the lipid extracts were combined and dried in OH mode in a SpeedVac. The dried samples were stored at −80 °C pending further analysis. Lipidomic analysis was performed using ExionLC‐AD‐Sciex QTRAP 6500 PLUS.^[^ ^59^ ^]^ MRM‐targeted quantitative techniques were established for the analysis of various polar lipids for comparative analysis and quantification of lipids by adding internal standards.

### Oxidized PUFA Analysis

Approximately 200 mg of mouse colonic tissues were extracted in a buffer comprising methanol containing 0.1% (w/v) of butylated hydroxytoluene and butylated hydroxyanisole with formic acid and internal standard cocktail added. Samples were vortexed to allow thorough mixing. A fixed number of ceramic beads pre‐cleaned with methanol was then added and the tissue samples were incubated at 1500 rpm for 12 h at 4 °C to ensure efficient extraction of eicosanoids from the tissue matrix. The samples were centrifuged at 4 °C for 10 min at 12 000 rpm, and the supernatant was extracted. The extraction was repeated for a second round. The pooled supernatants were enriched for eicosanoids via solid phase extraction (SPE) using Oasis Prime HLB columns (30 mg, Waters, USA).^[^ ^60^ ^]^ SPE eluents were transferred to tubes containing 20 µL of ethanol: glycerol 1:1 (v/v) to prevent complete desiccation, and dried under a flowing stream of nitrogen gas. The dried extract was re‐constituted immediately in 50 µL of water: acetonitrile: formic acid 63:37:0.02 (v/v/v) for mass spectrometric analysis. Eicosanoid analyses were conducted on a Shimadzu 40 × 3B‐UPLC coupled to Sciex QTRAP 6500 Plus (Sciex, USA). Eicosanoids were separated on a Phenomenex Kinetex‐C18 column (i.d., 100 × 2.1 mm, 1.7 µm) with mobile phases comprising (A) water: acetonitrile: formic acid 63:37:0.02 (v/v/v) and (B) acetonitrile: isopropanol 1:1 (v/v).^[^ ^61^ ^]^

### RNA‐Seq Analysis

Colorectal epithelial cells were isolated from the colorectum of four cKO mice and four littermate controls by incubation with 10 mm EDTA and 10 mm HEPES in PBS for 20 min at 37 °C. Epithelial fractions were collected by vigorous shaking and filtered through a 100‐µm cell strainer (BD Biosciences, USA). RNA was collected using the standard procedure described above, and RNA integrity was evaluated by using the RNA Nano 6000 assay kit on a Bioanalyzer 2100 system (Agilent Technologies, USA). RNA samples were sent to the Illumina NovaSeq 6000 platform of Novogene Technology for library preparation and sequencing. Data were analyzed on the free Novogene Cloud Platform (https://magic.novogene.com).

### Cell Culture

NCM460 cells and HT29 cell lines (generously gifted by Zhengquan Yu's lab at China Agricultural University) were cultured in RPMI 1640 supplemented with 10% FBS. HCT116 cell line was purchased from ATCC and cultured in McCoy's 5A supplemented with 10% FBS. All cell lines were tested and confirmed to be free of mycoplasma infection.

Small interfering RNAs (siRNAs) were purchased from Yaoyuan Biotechnology Co., Ltd. Cells were transfected with *SELENOI* siRNA or an equal amount of siRNA negative control for 24 h using Lipofectamine 3000 (L3000015, Invitrogen) for subsequent assays according to manufacturer's instructions. The siRNA sequence is summarized in Table S3 (Supporting Information).

The shRNAs were cloned into the pLV‐U6 vector, and shRNA vectors were cotransfected with pMDLg, pRSV rev, and pMG2.D (at a ratio of 4:3:2:1) into 293T cells to produce lentiviral particles, which were then transfected into HCT116 cells. After 72–96 h, cells were collected for further analysis or experiments. The shRNA sequence is summarized in Table S4 (Supporting Information).

Constructs of full‐length cDNAs of human *SELENOI* and *GPX4* were cloned into the pLV‐CMV‐puro vector, and overexpression vectors were cotransfected with pMDLg, pRSV rev, and pMG2.D (at a ratio of 4:3:2:1) into 293T cells to produce lentiviral particles, which were then transfected into HCT116 cells followed with puromycin selection.

### CRISPR Gene Editing

The synthesized sgRNA and Cas9 protein were incubated together to form ribonucleoprotein (RNP) complexes, resulting in active Cas9‐sgRNA complexes. Subsequently, these RNP complexes were introduced into HCT116 cells via electroporation, followed by the preparation of single‐cell clones using the limiting dilution method. Finally, the *SELENOI*‐deficient cell clone was identified by DNA sequencing and Western blotting.

### Western Blotting

Cell lysates were subjected to Western blotting according to standard procedures. Briefly, separated on a 6–12% SDS‐PAGE gel and transferred to a PVDF membrane (IPVH00010, MerckMillipore). The PVDF membrane was blocked with 5% skim milk powder for 1 h 30 min and incubated with primary antibody at 4 °C overnight. The results were acquired with a chemiluminescence imaging system (Sagecreation, China). Antibodies were applied as follows: anti‐GAPDH (AF2819, Beyotime), anti‐β‐actin (AF2811, Beyotime), anti‐α‐tubulin (11224‐1‐AP, Proteintech), anti‐*Selenoi* (ab194554, Abcam), anti‐Pla2g2a (ab23705, Abcam), anti‐Pla2g5 (sc‐393606, Santa Cruz), anti‐Alox15 (ab244205, Abcam), anti‐4HNE (ab46545, Abcam), anti‐Gpx4 (ab125066, Abcam) and anti‐Facl4 (ab155282, Abcam).

### Cell Viability Assay

Cell viability was assessed using the Enhanced Cell Counting Kit‐8 (C0042, Beyotime) according to the manufacturer's instructions. Cells were seeded into 96‐well plates (5000 cells well^−1^) and treated with RSL3 (S8155, Selleck), Erastin (S7242, Selleck) or dimethyl sulfoxide (D2650, Sigma) for 24 h post‐transfection, and each well was replaced with 100 µL of fresh complete medium containing 10 µL of CCK‐8 solution, then the cells were incubated at 37 °C, 5% CO_2_ for 1 h. The absorbance at 450 nm was measured with a luminescence detector (Bio‐Tek Synergy NEO).

### GSSG/GSH Assay

The GSSG and GSH levels were measured with a GSSG/GSH assay kit (G263, Dojindo) according to the manufacturer's instructions. The amount of GSH was derived by subtracting the amount of GSSG from the amount of total GSSG/GSH.

### Lipid ROS, Liperfluo, and FerroOrange Staining

The BODIPY 581/591 C11 probe (D3861, Thermo, 5 µm), the Liperfluo probe (L248, Dojindo, 1 µm), and the FerroOrange probe (F374, Dojindo, 1 µm) were added to each well, and the cells were incubated for 30 min at 37 °C. Then, the cells were fixed with 10% formalin solution and stained with DAPI. Photographs were taken at specific wavelengths for observation in a fluorescence microscope.

### ROS Assay

HT29 cells were cultured in confocal dishes and at the end of the treatment the cells were assayed with the Reactive Oxygen Detection Kit (S0033S, Beyotime) according to the instructions of the reagent supplier.

### Mitochondrial Membrane Potential and Apoptosis Assay

HT29 cells were inoculated in confocal dishes and assayed using the Mitochondrial Membrane Potential and Apoptosis Assay Kit (C1071M, Beyotime) at the end of cell treatment according to the manufacturer's instructions.

### Bioinformatics Analysis

The *SELENOI* expression levels in colorectal (Tumor: 455; Normal: 820), rectal (Tumor: 165; Normal: 789) and gastric cancer tumors (Tumor: 375; Normal: 391) from TCGA (https://www.cancer.gov/ccg/research/genome‐sequencing/tcga/) and GEO (https://www.ncbi.nlm.nih.gov/geo/) RNA‐Seq databases. The *Selenoi* expression levels in mouse colorectal epithelial cells from the mouse single‐cell sequencing database Tabula Muris (https://tabula‐muris.ds.czbiohub.org/).

### Statistical Analysis

The data were presented as the mean ± standard deviation (SD) unless otherwise stated and all analyses in this study were performed at least in triplicate. All microscope images were quantified with Image J software (National Institutes of Health, USA). The *P* value was obtained by unpaired two‐tailed Student's *t*‐test, and asterisks denote statistical significance (*P < 0.05; **P < 0.01; ***P < 0.001).

## Conflict of Interest

The authors declare no conflict of interest.

## Author Contributions

X.H., X.Y., and M.Z. contributed equally to this work. J.H. and C.L. designed the research. X.H., X.Y., M.Z., T.L., and K.Z. performed research. X.H., X.Y., and M.Z. analyzed data. X.H. and X.Y. wrote the manuscript. Y.D., X.L., Z.Y., C.L., and J.H. modified the manuscript. All authors read and approved the final manuscript.

## Supporting information

Supporting Information

## Data Availability

The data that support the findings of this study are available from the corresponding author upon reasonable request.
