# Supplementary material for: SELENOI Functions as a Key Modulator of Ferroptosis Pathway in Colitis and Colorectal Cancer
Source: Adv Sci (Weinh). 2024 May 17;11(28):2404073. doi: 10.1002/advs.202404073 (PMC11267378; doi:10.1002/advs.202404073)
Supplement: Supplementary file 1 — Supporting Information [file ADVS-11-2404073-s001.docx]

**Supporting Information**

SELENOI functions as a key modulator of ferroptosis pathway in colitis and colorectal cancer

*Xin Huang^1^, Xu Yang^1^, Mingxin Zhang^1^, Tong Li, Kongdi Zhu, Yulan Dong, Xingen Lei, Zhengquan Yu, Cong Lv* and Jiaqiang Huang**

**Figure S1.** Generating intestinal epithelium-specific *Selenoi* conditional knockout mouse. A) *In situ* hybridization for *Selenoi* in adjacent normal tissues and AOM-DSS colon tumors from mice. A represents adjacent tissues of tumor; T represents tumor. Scale bar: 50 μm (left and right panel) and 100 μm (middle panel). n= 3. B) Schematic diagram of the *Selenoi* knockout region. C) Strategy of generating cKO mice. D) Graph of genotype identification results of cKO mice. E) The expression of *Selenoi* in colonic tissues were detected by RNAscope from control and cKO mice. Scale bar: 50 μm. n= 3.

**Figure S2.** No apparent phenotype was observed in *Selenoi* cKO mice at homeostasis. A, B) Immunohistochemical staining results of P-Stat3, Cleaved caspase3 and Muc2 in jejunum (A) and colon (B) of control or cKO mice. Scale bar: 100 μm (jejunum) and 50 μm (colon). n= 3.

**Figure S3.** *Selenoi* deficiency in epithelial cells inhibited tumor growth. A) Schematics of generating AOM–DSS mouse colon tumor model. B) Quantification of body weight changes in control (n = 9) and cKO (n = 7) mice during tumor development. C) Gross images of AOM–DSS tumors in control (n = 9) and cKO (n = 7) mice. D) Quantification of number of polyps and polyp load in control (n = 9) and cKO (n = 7) mice. Number of polyps: total number of polyps/mice; polyp load: sum of diameters of all polyps. E) Histological images of colon tumors from control and cKO mice. Scale bar: 2 mm. F) Immunofluorescence detection of Selenoi in colon tumors from control and cKO mice. Scale bar: 50 μm. n = 5. G) Double immunofluorescence for β-catenin and Ki67 in colon tumors from control and cKO mice. Scale bar: 50 μm. n = 4. H) Gross images of xenograft tumors 2 weeks after transplantation with normal HCT 116 cells (NC) and SELENOI deficient (KO) HCT116 cells. I) Quantification for tumor volume shown in panel H. n = 6. The data are expressed by mean ± SD. ***P* < 0.01; ****P* < 0.001.

**Figure S4.** Lipidomic detection of phosphatidylethanolamine (PE) and phosphatidylcholine (PC) in colonic tissues from control and cKO mice. A) Differential expression analysis results revealed the upregulated and downregulated genes in colonic tissues of control and cKO mice, respectively. B) The Kennedy pathway. C) PCA plot showing the distribution of test results for the control and cKO groups. D) Heatmap for total PC in colonic tissues of control and cKO mice. n = 6. E) Heatmap for total PE in colonic tissues of control and cKO mice. n = 6. F) The PE/PC ratio of colonic tissues from control and cKO mice. n = 6. G) qRT-PCR analysis of altered ether lipids metabolism-related genes in HT29 cells after *SELENOI* siRNA transfection. n = 5. The data are expressed by mean ± SD. **P* < 0.05; ***P* < 0.01.

**Figure S5.** *Selenoi* deficiency causes ferroptosis *in vitro* and *in vivo*. A) qRT-PCR analysis validates altered ferroptosis-related genes in isolated colonic epithelial cells of control and cKO mice. n = 4. B) Western blotting for ALOX15, ACSL4 and 4-HNE in normal HCT 116 cells (NC) and SELENOI deficient (KO) HCT 116 cells. β-Actin was used as loading control. C) qRT-PCR analysis of *Cox2* in colonic tissues of control and cKO mice at 5+3 days after DSS treatment. n = 4. D) qRT-PCR for *Alox15* in AOM-DSS colon tumors from control and cKO mice. n= 5. E, F) Immunohistochemistry for Alox15, Acsl4 and 4-HNE in colonic tissues from control and cKO mice at 5+4 days after DSS treatment (E) and AOM-DSS colon tumors (F). Percentage of Alox15^+^ cells, Acsl4^+^ cells and 4-HNE^+^ cells were quantified. Scale bar: 50 μm. n = 5. The data are expressed by mean ± SD. ***P* < 0.01; ****P* < 0.001

**Figure S6.** *SELENOI* inhibition enhances ferroptosis sensitivity *in vitro*. A, B) Cell viability analysis of NCM460 cells (A) and HT29 cells (B) transfected with *SELENOI* siRNA upon 0-1 μM RSL3 treatment for 24 h. n = 5. C) ROS staining of HT29 cells transfected with *SELENOI* siRNA upon RSL3 treatment (1 μM for 24 h). Scale bar: 25 μm. n = 4. NC stands for negative control, cells without any treatment; PC stands for positive control, cells after treatment with the Rosup. D) Double immunofluorescence for Annexin V and Mito-Tracker in HT29 cells transfected with *SELENOI* siRNA upon RSL3 treatment (1 μM for 24 h). Scale bar: 50 μm. n = 4. NC stands for negative control, cells without any treatment; PC stands for positive control, apoptosis was induced by treatment of cells with TNF-α+SM-164. E) qRT-PCR analysis of ferroptosis-related genes in NCM460 cells after *SELENOI* siRNA treatment. n = 3. F) Representative immunohistochemical images for Gpx4 in colonic tissues from control and cKO mice. Scale bar: 50 μm. n = 3. The data are expressed by mean ± SD. **P* < 0.05; ***P* < 0.01; ****P* <0.0001.

**Figure S7.** The expression pattern of PLA2G5, PLA2G2A, ALOX15 in human colorectal cancer tissues. A-C) Immunohistochemical staining for PLA2G5 (A), ALOX15 (B) and PLA2G2A (C) in CRC tissue and adjacent normal tissues. Percentage of PLA2G5 ^+^ cells, ALOX15 ^+^ cells and PLA2G2A ^+^ cells were quantified. Scale bar: 100 μm. n = 10. The data are expressed by mean ± SD. ***P* < 0.01; ****P* <0.0001.

**Table S1.** Expression levels of selenoproteins in tumors of CRC patients

| Name | logFC | AveExpr | t | P.Value | adj.P.Val | B |
| --- | --- | --- | --- | --- | --- | --- |
| SELENO I | -0.8279 | 7.3161 | -8.3321 | 2.37E-10 | 5.46E-09 | 13.4529 |
| SELENO P | 1.9362 | 9.1330 | 7.1882 | 9.07E-09 | 8.29E-08 | 9.8371 |
| GPX2 | -0.9652 | 8.6605 | -7.1337 | 1.08E-08 | 8.29E-08 | 9.6623 |
| GPX3 | 0.5189 | 7.3049 | 6.4062 | 1.16E-07 | 6.64E-07 | 7.3174 |
| SELENO N | -0.3809 | 8.7740 | -5.7025 | 1.15E-06 | 5.31E-06 | 5.0460 |
| TXNRD3 | -0.2623 | 5.5928 | -5.1215 | 7.62E-06 | 2.92E-05 | 3.1912 |
| DIO2 | -0.3775 | 5.0996 | -4.7813 | 2.27E-05 | 7.46E-05 | 2.1241 |
| SELENO S | 0.3389 | 7.9176 | 4.7096 | 2.85E-05 | 8.20E-05 | 1.9017 |
| TXNRD1 | -0.3376 | 7.6389 | -4.3371 | 9.20E-05 | 0.0002 | 0.7628 |
| SEPHS2 | 0.1804 | 8.5034 | 3.5932 | 0.0009 | 0.0020 | -1.3965 |
| GPX4 | -0.2485 | 9.1641 | -3.4990 | 0.0011 | 0.0024 | -1.6560 |
| DIO1 | 0.1156 | 5.9797 | 3.4646 | 0.0013 | 0.0024 | -1.7498 |
| GPX6 | 0.2228 | 5.3558 | 3.4018 | 0.0015 | 0.0027 | -1.9199 |
| GPX1 | -0.1528 | 8.2223 | -3.2454 | 0.0023 | 0.0038 | -2.3355 |
| TXNRD2 | 0.0864 | 7.3230 | 3.0800 | 0.0037 | 0.0057 | -2.7626 |
| SELENO O | 0.1055 | 7.3444 | 2.7613 | 0.0086 | 0.0123 | -3.5448 |
| SELENO W | 0.1786 | 7.8365 | 2.7112 | 0.0098 | 0.0132 | -3.6627 |
| SELENO T | -0.1586 | 7.9139 | -2.5771 | 0.0137 | 0.0175 | -3.9704 |
| SELENO V | 0.1166 | 6.6381 | 2.4145 | 0.0203 | 0.0246 | -4.3283 |
| SELENO M | 0.0928 | 7.0062 | 2.3111 | 0.0259 | 0.0298 | -4.5469 |
| MSRB1 | -0.0819 | 7.9741 | -2.0075 | 0.0513 | 0.0562 | -5.1451 |
| DIO3 | 0.0928 | 6.1062 | 1.3621 | 0.1806 | 0.1888 | -6.1770 |
| SELENO K | 0.0189 | 6.3937 | 0.2672 | 0.7907 | 0.7907 | -7.0638 |

**Table S2.** Colorectal cancer tissue microarray information

| Pateint ID | Sex | Age | AJCC | T | N | N | M | Histology Grade | Histology grade description |
| --- | --- | --- | --- | --- | --- | --- | --- | --- | --- |
| 523001 | Male | 70 | IIB | 4 | 0 | 0/13 | 0 | 2 | ulcerative, moderately differentiated adenocarcinoma |
| 522851 | Female | 60 | IIB | 4 | 0 | 0/15 | 0 | 2 | ulcerative, moderately differentiated adenocarcinoma |
| 522408 | Female | 64 | IIB | 4 | 0 | 0/12 | 0 | 2 | ulcerative, moderately differentiated adenocarcinoma |
| 523172 | Female | 74 | IIIa | 2 | 1 | 3 (16) | 0 | 2 | ulcerative, moderately differentiated adenocarcinoma |
| 523417 | Male | 64 | IIA | 3 | 0 | 0/36 | 0 | 2 | ulcerative, moderately differentiated adenocarcinoma |
| 522258 | Male | 49 | IIA | 3 | 0 | 0/12 | 0 | 2 | Uplift, moderately differentiated adenocarcinoma, mucinous adenocarcinoma |
| 523713 | Male | 62 | IIIa | 3 | 1 | 1 (19) | 0 | 2 | ulcerative, moderately - poorly differentiated adenocarcinoma |
| 522858 | Female | 73 | IIIb | 4 | 1 | 1 (23） | 0 | 2 | ulcerative, moderately differentiated adenocarcinoma |
| 523262 | Male | 59 | IIIb | 4 | 1 | 2 (1) | 0 | 2 | ulcerative, moderately differentiated adenocarcinoma |

**Table S3.** siRNA sequence of *SELENOI*

| Name | Sequence (5'-3') | Sequence (5'-3') |
| --- | --- | --- |
| si*SELENOI-*260 | GCUUUCUGCUGGUCGUAUUdTdT | AAUACGACCAGCAGAAAGCdTdT |
| si*SELENOI*-713 | GGUAUGAACCUUUCCUGUUdTdT | AACAGGAAAGGUUCAUACCdTdT |
| si*SELENOI-*1055 | GGUUGCUGGUUCCUCUCUUdTdT | AAGAGAGGAACCAGCAACCdTdT |

**Table S4.** shRNA sequence

| Name | Sequence |
| --- | --- |
| *ALOX15*shRNA1 | GCTATCAAAGACTCTCTAAATCTCGAGATTTAGAGAGTCTTTGATAGC |
| *ALOX15*shRNA2 | TAGATGACTTCAACCGGATTTCTCGAGAAATCCGGTTGAAGTCATCTA |
| *ALOX1*5shRNA3 | GAAACTGGAAGGACGGGTTAACTCGAGTTAACCCGTCCTTCCAGTTTC |
| *PLA2G2A*shRNA1 | GAAACAAGACGACCTACAATACTCGAGTATTGTAGGTCGTCTTGTTTC |
| *PLA2G2A*shRNA2 | GATCAAGTTGACGACAGGAAACTCGAGTTTCCTGTCGTCAACTTGATC |
| *PLA2G2A*shRNA3 | GCTGTGTCACTCATGACTGTTCTCGAGAACAGTCATGAGTGACACAGC |
| *SELENOI*shRNA1 | GCCTGAGCTTTGGGAGAATATCTCGAGATATTCTCCCAAAGCTCAGGC |
| *SELENOI*shRNA2 | GCAGGCATAGTTTCCACTTTACTCGAGTAAAGTGGAAACTATGCCTGC |
| *SELENOI*shRNA3 | ACCTATTCACTGCAATGATTACTCGAGTAATCATTGCAGTGAATAGGT |
| *PLA2G5*shRNA1 | ATCTGGTGTATGGGTATTAAACTCGAGTTTAATACCCATACACCAGAT |
| *PLA2G5*shRNA2 | TACGCAAGAAGAGCCAAATTGCTCGAGCAATTTGGCTCTTCTTGCGTA |
| *PLA2G5*shRNA3 | TCGCACACAGTCCTACAAATACTCGAGTATTTGTAGGACTGTGTGCGA |
| *GPX4*shRNA1 | GTGAGGCAAGACCGAAGTAAACTCGAGTTTACTTCGGTCTTGCCTCAC |
| *GPX4*shRNA2 | CTACAACGTCAAATTCGATATCTCGAGATATCGAATTTGACGTTGTAG |
| *GPX4*shRNA3 | GCACATGGTTAACCTGGACAACTCGAGTTGTCCAGGTTAACCATGTGC |
